# Response to the Letter to the Editor Entitled “Focusing on Older Adults With Antineutrophil Cytoplasmic Autoantibody Negative Pauci-Immune Glomerulonephritis”

**DOI:** 10.1016/j.ekir.2025.03.035

**Published:** 2025-03-24

**Authors:** Lauren Floyd, Adam D. Morris, Mohamed Elsayed, Ajay P. Dhaygude, Matija Crnogorac, Andreas Kronbichler

**Affiliations:** 1 Division of Cardiovascular Sciences, The University of Manchester, Manchester, UK; 2 Renal Department, Royal Preston Hospital, Lancashire Teaching Hospitals NHS Foundation Hospitals, Lancashire, UK; 3 Department of Nephrology and Dialysis, Dubrava Clinical Hospital, Zagreb, Croatia; 4 Department of Internal Medicine, Nephrology and Hypertension, Medical University Innsbruck, Innsbruck, Austria

The Author Replies:

We appreciate the comments in the letter to the editor regarding our recent study, “Clinical Presentation and Outcomes of ANCA-Negative Pauci-Immune Glomerulonephritis.”^1^ The letter comments on the lack of subgroup analysis for older patients diagnosed with ANCA-negative pauci-immune glomerulonephritis (PIGN)^2^ and the role of frailty in disease outcomes, which raises important considerations for future research.

Our study aimed to investigate the clinical presentation, histopathological findings, treatment practices, and outcomes of patients with ANCA-negative PIGN. Although we noted that ANCA-negative patients were younger than those with ANCA-positive PIGN (56.6 vs. 63.6 years, *P* < 0.001), we acknowledge that further stratification by age, would provide valuable insight. Given that the incidence of ANCA-positive vasculitis increases with age because of factors such as immune dysregulation, understanding whether similar mechanisms contribute to ANCA-negative disease is of interest. A key limitation of this analysis is the relatively small number of older patients with ANCA-negative PIGN within our cohort. We present the crude data in Table 1; however, it is essential to emphasise that assessing older age and frailty’s impact on outcomes was beyond the scope of our study because of the limitations inherent with retrospective data collection. Consequently, any analysis of older patients is based on a small sample size, limiting statistical power and interpretability, and no definitive conclusions regarding frailty or age-related outcomes should be drawn from this small subgroup analysis.

**Table 1:** Demographics and outcomes associated with ANCA-negative PIGN patients aged 65 years and over

Patient profile	ANCA-negative PIGN aged ≥ 65 yrs *N* = 47
Sex, male: female (*n*)	27:20
KRT at diagnosis, *n* (%)	21 (44.7)
KRT recovery, *n* (%)	4 (19)
ESKD at 1 yr, *n* (%)	16 (34)
ESKD at 3 yr, *n* (%)	17 (36.2)
Mortality at 1 yr, *n* (%)	5 (10.6)
Mortality at 3 yr, *n* (%)	7 (14.9)
Induction treatment, *n* (%)	45 (95.7)

ANCA, antineutrophil cytoplasmic autoantibodies; ESKD, end-stage kidney disease; KRT, kidney replacement therapy; PIGN, pauci-immune glomerulonephritis.

Although the term frailty is often used synonymously with old age, the 2 are different, and frailty can occur in younger individuals as well. In addition, outcomes of a frail person developing vasculitis may differ from those of a robust person becoming frail because of vasculitis. Although frailty assessments were not included in our study, emerging evidence suggests that frailty is a crucial factor influencing disease progression, treatment responses, and morbidity in ANCA-associated vasculitis. Frailty measures are seldom used and have been largely unvalidated in this complex cohort. Incorporating frailty into prospective studies on ANCA-negative PIGN could offer valuable insights for improving risk stratification and guiding personalised treatment.

We appreciate this opportunity to discuss these important aspects and agree that additional research focusing on older adults with ANCA-negative PIGN, including measurements of frailty and age-stratified outcomes, would be beneficial. We encourage collaborative efforts to address these gaps and refine management strategies for such patient subgroups.
